# Floral Volatile Organic Compounds and a List of Pollinators of *Fallopia baldschuanica* (Polygonaceae)

**DOI:** 10.3390/insects13100904

**Published:** 2022-10-05

**Authors:** Anna Jakubska-Busse, Mariusz Dziadas, Iwona Gruss, Michał J. Kobyłka

**Affiliations:** 1 Department of Botany, Faculty of Biological Sciences, University of Wroclaw, 50-328 Wroclaw, Poland; 2 Faculty of Chemistry, University of Wroclaw, 50-353 Wrocław, Poland; 3 Department of Plant Protection, Wroclaw University of Environmental and Life Sciences, 50-363 Wroclaw, Poland

**Keywords:** *Fallopia aubertii*, Bukhara fleeceflower, floral scent, HS-GC/MS, VOCs

## Abstract

**Simple Summary:**

*Fallopia baldschuanica* (Polygonaceae) is an Asian plant growing wild in parts of Europe and North and Central America as an introduced taxon. Although *F. baldschuanica* is considered a potentially invasive alien plant species, little is known about its pollination biology in climatic conditions in Europe. In this study, we identified the volatile organic compounds emitted from *F. baldschuanica* flowers, from which some are important insect attractants. We also described the pollinator populations of this plant. We confirm that the chemical composition of floral aroma in *F. baldschuanica* attracts a large group of potential pollinators, which in addition to the intensive growth of the plant is a feature enabling the species to rapidly expand.

**Abstract:**

*Fallopia baldschuanica* (Polygonaceae) is an Asian plant growing wild in parts of Europe and North and Central America as an introduced taxon, in many countries it is considered a potentially invasive species. This article presents the list of 18 volatile organic compounds (VOCs) emitted by the flowers of *F. baldchuanica* and identified by headspace gas chromatography/mass spectrometry (HS-GC/MS) analyzes, and a list of flower-visiting and pollinating insects that have been observed in the city center of Wrocław (SW Poland). β-ocimene, heptanal, nonanal, α-pinene, 3-thujene, and limonene, were detected as the floral scent’s most important aroma compounds. *F. baldschuanica* also produces the aphid alarm pheromones, i.e., β-farnesene and limonene, that repels aphids. Additionally, the pollinators of *F. baldschuanica* were indicated, based on two years of observations in five sites in the urban area. It was found, that the pollinators of this plant with the highest species stability are: Diptera from families Syrphidae (*Chrysotoxum* *bicinctum*, *Eristalis* *pertinax*, *Eupeodes* *corollae*, *Episyrphus* *balteatus*, *Eristalis tenax*, *Syrphus ribesii*, *Eristalis intricaria*), Muscidae (*Musca domestica*), Sarcophagidae (*Sarcophaga* spp.), Calliphoridae (*Lucilia sericata*, *Lucilia caesar*), Hymenoptera from families Vespidae (*Vespula vulgaris*), and Apidae (*Apis* sp., *Bombus* sp.). The key role of VOCs in adaptation to plant expansion is discussed.

## 1. Introduction

*Fallopia baldschuanica* (Regel) Holub (syn. *Bilderdykia baldschuanica* (Regel) D.A.Webb, *Fagopyrum baldschuanicum* (Regel) Gross, *Fagopyrum baldschuanicum* H. Gross, *Polygonum baldschuanicum* Regel*, Reynoutria baldschuanica* (Regel) Moldenke) [1] also known as “Bukhara fleeceflower”, “Russian Vine”, “Fleece Flower”, “Fleece Vine”, “China Fleece Vine”, “Silver Fleece Vine”, “Silverlace Vine”, or “Mile—a minute Plant” is a woody deciduous climber that belongs to the Knotweed family [2,3]. The taxonomic status of this plant is unclear. Currently*, F*. *baldschuanica* is treated as a synonym of *Fallopia aubertii* [4,5,6], although until recently these species had been separated as a different taxon [7,8]. The plant is native to Asia, mainly distributed in China, Russia, Kazakhstan, Afghanistan, Tajikistan, Pakistan, and Iran [7,9,10]. This species was probably introduced into Europe from Baldshuan Khanate in Turkestan [11]. In the wild, plants grow at altitudes from 500 (900) m to 3200 m a.s.l. [7,12]. *F. baldschuanica* is also used in traditional and folk medicine to treat fever, pneumonia, and gout [13].

*Fallopia baldschuanica* is grown as an ornamental plant, that is often used by architects in Europe due to its extremely vigorous growth habit, as a cover for ugly structures, unsightly fences, and other garden structures. It often occurs as discarded material on waste ground, but until recently it was thought to be rarely well naturalized [14,15]. It can be found growing wild in parts of Europe and North and Central America [9]. It was first recorded outside cultivation in 1942 in the sea dunes in Duinbergen (Knokke, Belgium) [16]. This alien species was also classified as “thugs” by the Royal Horticultural Society (RHS) [17]. This term refers to garden plants that are easily available to buy, and that have the potential to become a nuisance [17]. Plants grow at a tremendous rate, and can put on over 4 m in one year; they may smother any other plants in their way [10].

Today, in many regions of Europe, *F. baldschuanica* is treated as an invasive plant, rapidly spreading beyond its intended borders [18]. *F. baldscuanica*, like other species of knotweeds, can reproduce sexually through seed production and/or clonally propagated by rhizomes and stem cuttings [19]. This species has been known to form a hybrid with *Fallopia japonica*, the hybrid was named *F.* × *conollyana* [19]. A characteristic feature of this species is the white, fragrant, long flowering (from July until the first frost) flowers, which provide nectar and pollen to various pollinating and visiting groups of insects.

Although *F. baldschuanica* is considered a potentially invasive plant species, little is known about its pollination biology in climatic conditions in Europe. Recently, scientists have been very interested in issues related to the hybridization of *Fallopia* species, important driving forces of invasive processes [19,20]. It should be remembered that pollinators play a key role in this process. Additionally, the occurrence of alien plant species can negatively affect the number of pollinators visiting native species. It was found that in urban areas pollinators choose more frequently the invasive plants, in comparison to similar pollinator communities in natural areas. Therefore, native plants in urban areas are less visited by insects and their diversity may decline in the future, which is a major aspect of its negative impact on the environment [21]. Additionally, pollinators could be more specialized in urban, than in natural areas [22].

In this study, we investigated: (i) what volatile organic compounds (VOCs) are emitted by *F. baldschuanica*; (ii) which groups of insects visit and pollinate plants of *F. baldschuanica*, in an urban area of Wrocław, Poland; as well as, based on data reported in the literature, (iii) what role the identified VOCs can play in the biology of the observed flower-visiting insects. 

## 2. Materials and Methods

### 2.1. Plant Material

**Figure 1:**
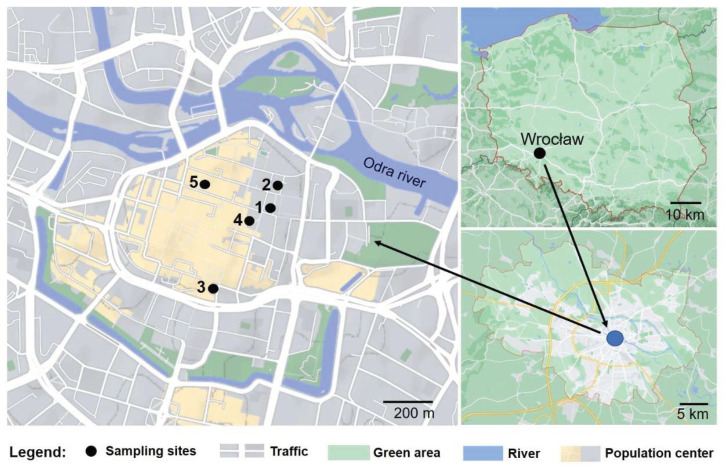
Location map of investigation area in the center of Wrocław: sampling sites: (1) 5 Kotlarska St., (2) 2–4 Jodłowa St., (3) 78–80 Szewska St., (4) Łaciarska St. (backyard), (5) 21 Kotlarska St.

Fully open flowers of *F. baldschuanica* at the same developmental stage were collected from individuals of the five populations (sites 1–5) located in the center of Wrocław city, between 7 and 24 October 2020, and were used for the chemical analyzes. The study sites were located in the Old Town of Wrocław (Figure 1). Plants grew on the fence (sites 1 and 4) and in the vicinity of the dumpsters (sites 2, 3, 5). The location of sampling sites with GPS (Global Positioning System) coordinates: (1) 51°06′40.0″ N 17°02′13.1″ E; (2) 51°06′43.3″ N 17°02′15.3″ E; (3) 51°06′27.5″ N 17°02′00.1″ E; (4) 51°06′38.8″ N 17°02′10.4″ E; (5) 51°06′43.6″ N 17°01′58.1″ E. The largest distance between the sites was 600 m.

### 2.2. HS-GC/S Analysis of Volatiles Fractions

The analysis of volatiles from the sample was carried out using GC-MS QP 2010 Ultra system (Shimadzu, Kyoto, Japan) equipped with headspace autosampler HS-20 (Shimadzu Corporation, Kyoto, Japan). A fresh sample of flowers (2 g) was weighed directly into a clean headspace vial (20 mL) with 10 mL pure water Merck Millipore (Merck Millipore, Warsaw, Poland) containing 180 ug 2-octanol (Sigma-Aldrich, Poznan, Poland) as internal standard, and closed using a screw cap with butyl septa (Sigma-Aldrich, Poznan, Poland). The sample was analyzed in triplicate. Table 1 shows the standard deviation (SD) of three replicates.

Program of head space autosampler: oven temperature 80°C, sample line 150°C, transfer line 150°C, equilibration time 10 min, pressurizing time 0.5 min (60 kPa), load time 0.5 min, injection time 1.00 min, needle flush time 2.00 min, shaking level 2. Chromatography analysis was carried out using ZB-5 ms capillary column (30 m × 0.25 diam., 0.25 film, Phenomenex, Torrance, CA, USA) with 1 mL/min flow of helium 6.0 purity (Linde Gas, Kraków, Poland) with split 1:2. Oven program: 40°C–0.00 min, 4°C/min to 140°C hold 0.00 min, 15 °C/min to 320°C hold 0.00 min. Single quadrupole mass detector operates in 38.00 to 488 scan range with 20,000 scan speed. The temperature of the ion source was 220°C, the interface was 260°C, and the solvent cut time was 1 min. The LabSolution ver 4.20 (Shimadzu, Kyoto, Japan) was used as software for data processing with NIST libraries 14 and 17 as databases. The tentative identification of compounds was based on a comparison with the mass spectral library and is presented in Table 1 and Supplementary Material S1.

The odor characteristics of the chemical compounds that are components of the scent of the analyzed plant, which were identified during the chromatographic study, were based on information available online [23].

### 2.3. Field Observations of Insects Activity

The observations were conducted during the flowering season (more precisely the peak of the plant flowering period) from the beginning of August to the end in October in 2019, and from the middle of July to the middle of October in 2020, in the urban area of Wrocław (SW Poland), in the five mentioned above sites (sites 1–5), located in Wrocław city center (Figure 1). Observations were made over a span of 2–6 h, covering daylight hours (9:00 a.m.–6:00 p.m.). Flower visitors were observed, with a total observation time of >60 h. The pollinators and visitor insects were photographed/documented using a Canon digital camera D50 camera (Canon EOS 50D, Canon Inc., Tokyo, Japan) with a Tamron 90 mm f /2.8 SP Di Macro lens, captured in field conditions by A.J-B. and identified by specialists. Only bumblebee species protected by law in Poland were not caught, they were photographed on flowers of *F. baldschuanica* and identified by entomologists on the basis of macrophotographs. 

The insect abundance and related ecological indices between sites were compared using the non-parametric Kruskal-Wallis test by ranks, performed in SAS University Edition. The analyzes were performed separately for 2019 and 2020. The following indices were calculated: the dominance index (d), the Berger-Parker dominance index (D) [24], the Shannon-Weaver index (H’) [25], the Pielou index (J) [26], Margalef’s species richness index (S) [27], species stability index C [28], and the Jaccard similarity index (SJ) [29]. The formulas used for calculations of the indices are included in the Supplementary Material S2.

The taxa abundance was correlated with the date, time of observation, and site, using the constrained analysis Canonical Correspondence Analysis (CCA). The analyzes were performed in Canoco 5.0. the significance of the axes was tested using the Monte-Carlo test. 

## 3. Results

### 3.1. Identification of VOCs

**Figure 2:**
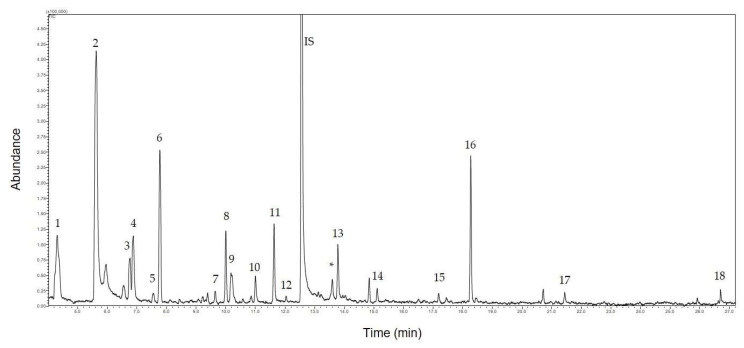
The chromatogram obtained during the analysis of the volatile organic compounds (VOCs) emitted by *Fallopia baldschuanica*. Abbreviation: *—unknown compound; IS—internal standard. The numbers in the chromatogram correspond to the numbers of the identified compounds in Table 1. Unnumbered signals are from the column filling (stationary phase) and/or from air pollution in the environment in which the flowers were packed into vials.

The analysis of volatiles from the sample was performed by headspace analysis from fresh flowers using gas chromatography coupled with mass spectrometry (HS-GC/MS). The obtained chromatogram is presented in Figure 2.

The list of identified volatile organic compounds (VOCs) together with its formulae and the odor characteristic of the identified compounds emitted by *F. baldschuanica* is presented in Table 1.

The analysis of volatiles from the sample reveals that *F. baldschuanica* emits mainly hydrocarbons: saturated (3, 8, and 15) and unsaturated commonly classified as monoterpenes (5, 6, 7, 10, 11, and 12) and sesquiterpene (18). Additionally, three aldehydes (1, 4, and 13), one unsaturated alcohol (2), and esters (9, 14, 16, and 17) having unsaturated chains are being produced.

A detailed analysis of the presented chromatogram reveals that there is another volatile compound present in the scent of *F. baldschuanica*. Its retention time is 13.60 min (abundance 18 ppm with SD—0.05). Unfortunately, we were not able to identify this chemical, thus its structure remains unknown.

### 3.2. True Pollinators and Visitors Insects

**Figure 3:**
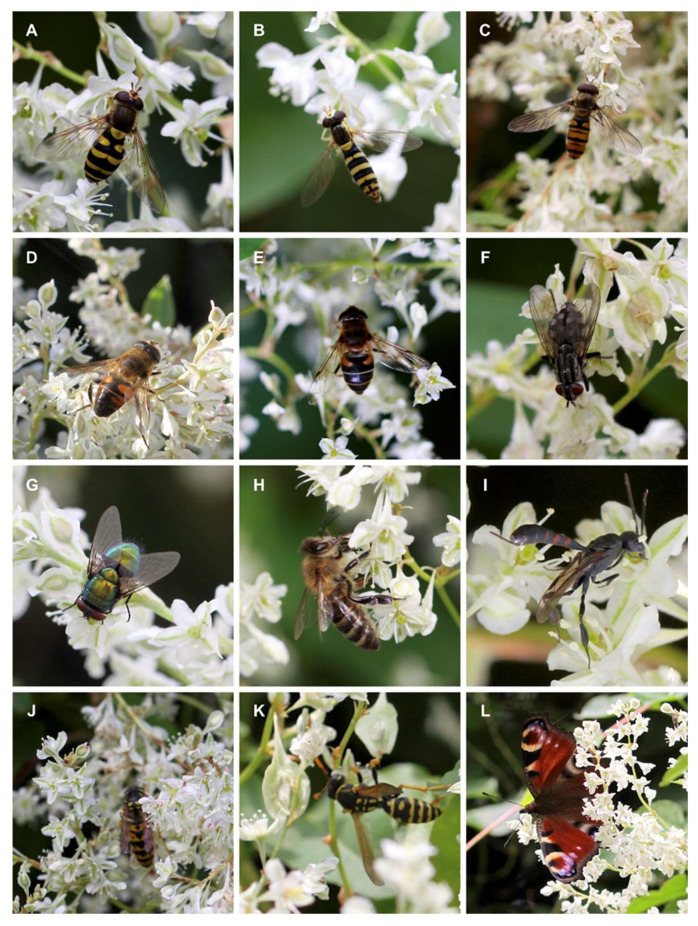
Pollinators and visitors of *Fallopia baldschuanica* (Polygonaceae) were observed in 2019–2020 in the center of Wrocław, SW Poland. (**A**) *Syrphus ribesii*, (**B**), *Sphaerophoria scripta*, (**C**) *Episyrphus balteatus*, (**D**) *Eristalis tenax*, (**E**) *Eristalis pertinax*, (**F**) *Sarcophaga* sp. (**G**) *Lucilia sericata*, (**H**) *Apis mellifera*, (**I**) *Gasteruption assectator*, (**J**) *Vespula vulgaris*, (**K**) *Polistes dominula*, and (**L**) *Aglais io*.

The analyzes were performed separately for 2019 and 2020. First, we aimed to find out if the pollinator’s pool changed during the research. Secondly, each season is characterized by different weather and vegetation conditions, and therefore it was more appreciated to analyze each season separately. The pollinator community was the same in both years of the study, accounting for 24 species. However, the specific population indices differ between the seasons and are further described for the season 2019 and 2020. Some of the pollinators observed during the two-year study period are presented in Figure 3.

2019: The number of plant visitors, as well as the number of species, differed significantly between sites (Table 2). The largest abundances and species numbers were found in sites 1 and 4. Analyzing the species diversity indices, the Margalef’s index and Shannon-Weaver index show better pollinator diversity in site 4 in comparison to measures in four other sites. The Pielou’s index, representing the species’ evenness, shows the lowest evenness for site 1, despite the large pollinator abundances. In total, 24 pollinator species were observed. At each site, four eudominants occurred, with the dominance index of >0.1. The Berger-Parker Dominance, which expresses the importance of the most abundant species, was the highest in site 4. There were several species which stability index (C) was > 100, taking into account all sites. Among those species observed in 2019 are Diptera from the families Syrphidae (*Chrysotoxum bicinctum*, *Eupeodes corollae*, *Episyrphus balteatus*, *Eristalis tenax, Syrphus ribesii)*, Muscidae *(Musca domestica)*, Calliphoridae (*Lucilia sericata*, *Lucilia caesar*), and Hymenoptera from the families Vespidae (*Vespula vulgaris*) and Apidae (*Apis* sp., *Bombus* sp.) (Table S1, Figure 3).

The CCA biplot shows the species abundance in relation to the date and time of observation, as well as the site (Figure S1 and Table S2). The total variance explained by the variables was 25.24 %, while mostly the CCA1 (variance explained = 16.28%) corresponds to the taxa abundance. It was found that taxa abundance decreases from the beginning (August) to the end (October) of the season. The community composition of pollinators of site 5 was the most universal, site 3 the most unique (Figure S1, Table 3).

2020: The total number of plant visitors was significantly higher in sites 3 and 4, while the species number was significantly lower in sites 4 and 5 in comparison to other treatments (Table 4). Additionally, the diversity indices, Margalef, Shannon-Weaver, and Pielou, show better biodiversity responses in sites 3 and 4 than in the remaining populations. Similar to 2019, 24 species were observed. There were 3–4 eudominants (species accounting for more than 0.1 of total species occurrence) observed on each site. The species, with the stability index accounting for more than 100 are: Diptera from the families Syrphidae (*Chrysotoxum bicinctum*, *Eristalis pertinax*, *Eupeodes corollae*, *Episyrphus balteatus*, *Eristalis tenax*, *Syrphus ribesii, Eristalis intricaria*), Muscidae (*Musca domestica*), Sarcophagidae (*Sarcophaga* spp.), Calliphoridae (*Lucilia sericata*, *Lucilia caesar*), and Hymenoptera from families Vespidae (*Vespula vulgaris*) and Apidae (*Apis* sp., *Bombus* spp.) (Table S1, Figure 3). The species with the highest stability index were similar in both years, except for *Sarcophaga* spp., in which stability increased in 2020. Analyzing the CCA biplot, the taxa were uniformly distributed along with the CCA 1 and CCA 2 (Figure S2, Table S3). The total variance explained by variables is 26.18%, while CCA 1 explained 9.93% and CCA 2 explained 7.44% of the variance. The similarity between sites was low. Similar to 2020, most of the species were negatively distributed over time (the taxa abundance decreased during the season going). The time of sampling during the day has a minor impact. In the second year of the observations, the species similarity between sites increased and the similarity index between all sites was more than 0.8 (Table 5).

## 4. Discussion

The intense floral scent in *F. baldschuanica*, detectable by the human nose, is composed of many interesting volatile organic compounds (VOCs) that can influence or manipulate insect behavior. The first of these is β-farnesene, one of two naturally occurring stereoisomers of this compound. Both (α- and β-) isomers are also insect semiochemicals, i.e., organic compounds used by insects to convey specific chemical messages that modify behavior or physiology. β-Farnesene is the most common isomer of the pair. It is found in the coating of apples, and other fruits, and it is responsible for the characteristic green apple odor [30]. It is a constituent of various essential oils, it occurs both in gymnosperms such as *Larix leptolepis* [31], and in several families of angiosperms, e.g., in Fabaceae: *Medicago sativa* [32]; in Asteraceae, *Anthemis tinctoria*, *Chamomilla recutita*, *C. suaveolens*, *Leucanthemum vulgare* [33]; and *Matricaria perforate*, in Lamiaceae, *Mentha aquatica* var. *citrata* [34]; as well as in the Cannabaceae family, *Cannabis* spp. [35]. Several plants, including potato species, have been shown to synthesize this semiochemical as a natural insect repellent [36,37], e.g., transgenic plants of *Arabidopsis thaliana* emitted this compound as a repellent to the *Myzus persicae* (Hemiptera, Aphididae) [38]. Furthermore, this compound is also widespread in the animal kingdom. For example, increased amounts of β-farnesene have been found in the urine of dominant male mice (*Mus domesticus*), which probably plays a role in marking the territory [39]. Several insect pheromones, including β-farnesene, were found in the urine of female African elephants (*Loxodonta africana*) [40].

This substance fulfills many tasks, especially in insects, for example as a pheromone in marking the nests of solitary bees belonging to the genus *Andrena* (Andreninae, Andrenidae) [41], as a defense allomone, and as a trace pheromone of the worker ant species *Myrmecia nigriceps* [42] or as kairomone for finding the prey in some predatory ground beetles (Coleoptera: Carabidae) [37,43]. It acts as an alarm pheromone in termites [44] or a food attractant for the apple tree pest, the codling moth [45]. Moreover, β-farnesene has been reported in the floral scent of a number of male euglossine bee-pollinated orchids [46,47]. This compound is a component of the sex pheromone of the medfly fly, *Ceratitis capitate* and may also be a pheromone component in a beetle [48]. Interestingly, its derivatives (E,E)-farnesol has frequently been reported as a component of the secretions of the Dufour’s glands of Andrenid bees, of the Nasonov glands of honey bee workers, of the labial glands of bumble bees, and of the mandibular glands of leaf-cutting ants [48]. Unfortunately, the role of this compound as an insect attractant in *Fallopia* spp. has yet to be proven.

Additionally, β-farnesene plays an important role in aphid behavior [36]. It is also released by greenflies as an alarm pheromone upon death to warn away other aphids [49]. This sesquiterpene is produced by many species of aphids and is a signal for nearby individuals to stop foraging and escape. Aphids are plant pests, they suck plant juices, feed on shoot juice, and usually feed on young, juicy apical shoots, as well as on young leaves, inflorescences, and flower buds, which can damage them. Alert pheromones, apart from repelling aphids, are often attractants to their natural enemies. We believe that the ability to produce this floral scent compound may be considered a beneficial adaptation of the pest elimination by *F. baldschuanica*. This hypothesis needs further examination. β-Farnesene is also reported as an oviposition stimulant [50] for the hoverfly *Episyrphus balteatus*, an insect that has been observed as a visitor and true pollinator of *F. baldschuanica* (Figure 3C).

A repellent for aphid nymphs of *Panaphis juglandis* and *Chromaphis juglandicola* [51] is limonene, another VOCs which has been identified in *F. baldschuanica*. The larvicidal activity effect also has another compound identified by us, i.e., γ-terpinene. This terpene is a component of essential oils of many plant species e.g., in the family Lamiaceae, in *Thymus vulgaris* and *Origanum* species it is also considered an effective repellent against mosquitoes [52]. An important scent compound emitted by *F. baldschuanica* is also hexanal, considered an insect attractant, among others for flies of Psilidae (Diptera) [53]. Additionally, other VOCs, i.e., limonene and β-ocimene that we detected in *F. baldschuanica,* have been reported as constituents of the volatile bouquet of several citrus species [54]. These compounds were identified as an ingredient of different infested fruit species that attracted other parasitoids, such as *Agathis bishopi* (Hymenoptera: Braconidae) and *Aphidius gifuensis* (Hymenoptera: Aphidiidae) [54]. In addition, the β-ocimene has very common plant volatiles released in significant amounts from the leaves and flowers of many plant species, and is a general attractant of a wide spectrum of pollinators [55], including the honeybees *Apis mellifera* and bumblebees (*Bombus* spp.) [56,57] that we have observed (Figure 3G–I, Table 2). This acyclic monoterpene can play several biological functions in plants, depending on the organ and the time of emission and potentially affecting floral visitors, and also by mediating defensive responses to herbivory [55]. Due to its attractive fragrance, β-ocimene may be one of the key compounds emitted by *F. baldschuanica* that lures pollinating insects, and also attracts natural enemies of the phytophagous. Besides, phytophagous insects can identify the β-ocimene, and use it as chemical cues to identify their host plants [58,59]. The presence of this floral aroma compound has not yet been reported in representatives of the genus *Fallopia*. The β-ocimene and limonene are also reported as predominant components of essential oils of species of many plant families [60]. The nonanal, another compound we identified in *F. baldschuanica,* attracts e.g., the observed by us as flower visitors, flesh flies (Sarcophagidae) [61], and was also attractive to the herbivorous beetle, *Hylastes opacus* (Coleoptera, Scolytidae), in North America [62]. Unfortunately, we did not observe the mentioned taxa of beetles visiting *F. baldschuanica*, but we identified beetles from other families, i.e., Cantharidae—*Cantharis pellucida* and Cerambycidae—*Gaurotes virginea*.

It is worth mentioning that heptanal, α-pinene, and limonene, have been isolated from flowers of the genus *Ophrys* [63], taxon pollinated by the sphecid and scoliid wasps and solitary bees, including long-horned bees from genus *Eucera* (Hymenoptera, Apidae). Orchids, especially of the *Ophrys* genus, belong to plants that are highly specialized in attracting specific groups of pollinators. Thus, it is possible that the high frequency of *F. baldschuanica* visitors of Apidae (*Apis mellifera*, *Bombus* spp.) is the result of the emission of these three volatile chemicals. Interestingly, neotropical orchids pollinated by the *Euglossini* (Hymenoptera, Apidae) emit a very intense fragrance but do not produce nectar or pollen food. Their flowers are pollinated by male euglossine bees, who are attracted by volatile semiochemicals, e.g., α-pinene and ocimene [63], and thus the compounds emitted by *F. baldschuanica*. Although we did not observe the *Euglossini* on *F. baldschuanica*, because these insects exclusively occur in South or Central America, but in the center of Wroclaw city, the flowers of *F. baldschuanica* are frequently visited by other bee taxa (*Apis* spp., *Bombus terrestris*). However, it seems that the synergistic effect of all VOCs identified in *F. baldschuanica* best explains the frequency of all groups of visitors and potential pollinator insects that we have observed. Moreover, based on our observations, the emission of a strong, perceptible odor by *F. baldschuanica* over a long period of the day, i.e., from about 10.00 am to sunset, results in the possibility of visiting a large group of insects and thus increases the chance of pollinating more flowers in inflorescences. Additionally, the flowers of this plant produce nectar from mid-July to the end of October (until the first frosts). We believe that the relatively long period of nectar production, and thus food provision for many groups of insects, may pose a key factor in a plant’s success and may ensure its invasion. *F. baldschuanica* produces smaller flowers of large amounts, constantly offering visiting insects access to food. Some researchers indicate that pollinators or other groups of beneficial insects (predators, parasitoids) recognize the host plant, not by single compounds, but by specific ratios of ubiquitous compounds [64,65]. Additionally, insects process the olfactory signals by the receptor neurons [61]. This should be taken into account in planning olfactory experiments on insect attractants. Field observations of the insects’ behavior have shown that during intensive flowering, i.e., from mid-July to mid-October 2019 and 2020, the plant emits a very intense scent and is visited by a large number of insects, although its activity changed seasonally. In total, 24 species were recognized, which is high diversity for an urban ecosystem. All the species found are native to Poland and central Europe. Based on our research, we are not able to access if the occurrence of this plant species decreased the number of pollinators visiting native plant species and how it affects native populations. The recent study of Kovács-Hostyánszki et al. [66] addresses this question, indicating that the populations of alien plant species in the long-term negatively affect native plant species and pollinator communities. Surprisingly, invasive plants can increase the foraging resources of pollinators, but only for the short term [66]. On the other hand, knowing that *F. baldschuanica* is a food base for many pollinator species, it may be considered as a beneficial plant for pollinators in the high-urbanized area, characterized by low plant diversity. Nonetheless, in designing greenery, the invasiveness of *F. baldschuanica* should be considered and native species, which are equally attractive to pollinators, should be introduced.

To compare another related plant species, i.e., Japanese knotweed *Reynoutria japonica* (syn. *Fallopia japonica*), it was visited by 14 pollinators [21]. The species diversity indices were low, which is rather specific for urban areas. Urbanization generally reduced pollinator diversity when compared to natural ecosystems [67]. Adult insects (imagines) of flies (Diptera), mainly representatives of Syrphidae, Muscidae, Sarcophagida, and Calliphoridae, as well Hymenoptera (*Vespula vulgaris*, *Apis* sp., *Bombus* sp.), were the most active pollinators of *F. baldschuanica* from July to even until mid-October. Kovács-Hostyánszki et al. [66] found, that mainly hoverflies benefit from plant invasion, which is in line with our studies. The same authors indicate that the number of wild bees decreased and the number of honey bees increased after plant invasions. Wild bees are often more closely associated with particular plant species and therefore their population is declining after being impoverished by the ecosystem [68].

We also observed differences between pollinator populations in the five study sites, which is probably also a result of the studied habitat specificity. The urban ecosystems are characterized by a high degree of habitat heterogeneity with microclimates and microhabitats variations [69]. However, in 2020, the species similarity between the five study sites was very high. We may therefore suspect that insect populations have mixed up over time. Interestingly, the results of our research confirm the general data on the pollination biology of related plant species, i.e., *R. japonica,* provided by Balough [70]. According to this author, the most frequent visitors of *R. japonica* flowers are syrphid flies (Diptera, Syrphidae) and muscid flies (Diptera, Muscidae). Additionally common are hymenopterans (Hymenoptera), beetles (Coleoptera), true bugs (Hemiptera, Rhynchota), moths, and butterflies (Lepidoptera) [70]. Among this rich list of insects, we have not only observed the bugs (Rhynchota) as visitors/potential pollinators of *F. baldschuanica*, but the mentioned bugs feed on plants, using the sucking and piercing mouthparts to extract plant sap. We have often observed these phytophagous insects near *F. baldschuanica* plants, but never on flowers. Additionally, in the study on other related species *R.* *japonica*, ants were classified as insect visitors [21].

## 5. Conclusions

Among the main fragrance components of *F. baldschuanica* floral scent, the most important are β-ocimene, heptanal, nonanal, α-pinene, 3-thujene, and the alarm pheromones, β-farnesene, and limonene. Emitting such strong attractants by this potential invasive plant explains the observed numerous groups of flower-visiting insects both Hymenoptera (*Apis* sp., *Vespula* sp.) and Diptera (Syrphidae, Calliphoridae, Muscidae). Based on the results obtained, we hypothesize that the chemical composition of floral aroma in *F. baldschuanica* is a key factor in this species’ evolution because volatile organic compounds (VOCs) attract a large group of potential pollinators.

## Figures and Tables

**Table 1:** List of volatile organic compounds (VOCs), its chemical structure, and odor characteristic identified in *Fallopia baldschuanica*. Abbreviation: RT—retention time/minutes; SD—standard deviation.

	Chemical Name	RT	µg/2 g Sample	SD	NIST Search [%]	Kovats Index	Structure
1	hexanal	4.31	0.66	0.08	90	824	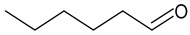
		Odor characteristic: green, fruity, acorn, tallowy, fishy, grassy, herbal, leafy					
2	hex-3-en-1-ol	5.67	7.44	0.56	95	857	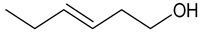
		Odor characteristic: fresh, green, grassy, leafy					
3	nonane	6.75	1.02	0.12	94	900	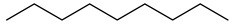
		Odor characteristic: fusel-like					
4	heptanal	6.87	1.66	0.19	94	904	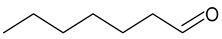
		Odor characteristic: citrus, green, fatty, dry fish, pesticide, solvent, smoky, rancid, fruity					
5	3-thujene (5-isopropyl-2-methylbicyclo[3.1.0]hex-2-ene)	7.55	0.24	0.04	92	928	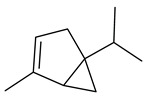
		Odor characteristic: woody, herbal, green					
6	α-pinene (2,6,6-trimethylbicyclo[3.1.1]hept-2-ene)	7.75	2.48	0.19	94	935	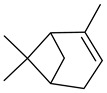
		Odor characteristic: terpeny, fruity, sweet, green, woody, pine, citrus, lime, camphor					
7	β -myrcene (7-methyl-3-methylene-1,6-octadiene)	9.65	0.16	0.03	92	991	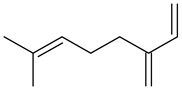
		Odor characteristic: metallic, musty, geranium, sweet, fruity, ethereal, soapy, lemon, spicy, woody					
8	decane	10	1.1	0.11	95	1000	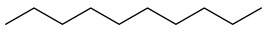
		Odor characteristic: fusel-like, fruity, sweet					
9	hex-3-ene-1-ol acetate	10.21	1.34	0.18	90	1008	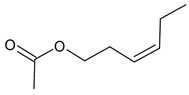
		Odor characteristic: green, freshly cut grass, slightly fruity					
10	limonene (1-methyl-4-(1-methylethenyl)-cyclohexene)	11.01	2.12	0.33	93	1036	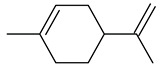
		Odor characteristic: licorice, green, citrus, ethereal, fruity					
11	β-ocimene ((E)-3,7-dimethyl-1,3,6-octatriene)	11.63	4.02	0.76	96	1056	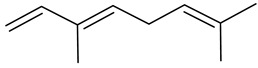
		Odor characteristic: herbal, mild, citrus, floral, woody, sweet, orange, lemon					
12	γ-terpinene (1-isopropyl-4-methyl-cyclohexa-1,4-diene)	12.05	0.06	0.01	90	1069	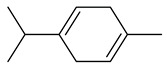
		Odor characteristic: citrus, terpeny, herbal, fruity, sweet					
13	nonanal	13.78	0.72	0.1	97	1118	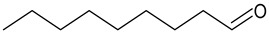
		Odor characteristic: gravy, green, tallowy, fruity, gas, chlorine, floral (rose, orris), waxy, sweet, melon, soapy, fatty, lavender, citrus fruit					
14	hex-3-ene-1-ol butanoate	15.12	0.52	0.1	97	1153	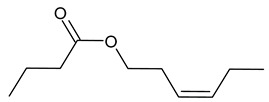
		Odor characteristic: sweet green, freshly cut grass, slightly fruity					
15	dodecane	17.18	0.16	0.03	93	1200	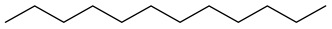
		Odor characteristic: fusel-like					
16	butanoic acid, 2-methyl, 3-hexenyl ester (3-hexen-1-yl 2-methyl butyrate)	18.19	16	2.05	96	1233	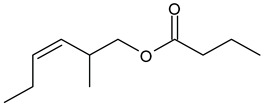
		Odor characteristic: fresh green apple sweet fruity pear					
17	2-butenoic acid, 2-methyl, (3Z)-3-hexen-1-yl	21.45	1.56	0.23	91	1325	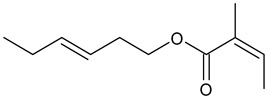
		Odor characteristic: leafy, green, vegetable					
18	β-farnesene ((E)-7,11-dimethyl-3-methylene-1,6,10-dodecatriene)	26.59	0.14	0.02	90	1458	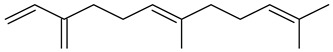
		Odor characteristic: woody, green					

**Table 2:** The species and community responses of pollinators observed on *Fallopia baldschuanica* flowers in 2019.

Site	1		2		3		4		5		Total	C
	n	d	n	d	n	d	N	d	N	d		
*Episyrphus balteatus*	150.80	0.13 *	29.78	**0.15**	7.00	0.04	202.40	**0.15**	87.89	**0.14**	219.62	**170.83**
*Sarcophaga* spp.	129.00	**0.11**	19.85	**0.17**	49.25	**0.34**	180.70	**0.13**	145.89	**0.25**	207.02	**191.67**
*Musca domestica*	149.70	**0.13**	26.47	**0.32**	37.75	**0.28**	164.10	**0.12**	121.44	**0.19**	195.06	**200.00**
*Chrysotoxum bicinctum*	97.40	**0.11**	0.00	0.00	0.00	0.00	110.00	0.08	52.75	0.07	172.14	**116.67**
*Apis* sp.	116.20	0.09	4.38	0.03	9.00	0.07	138.10	**0.10**	6.22	0.01	127.14	**170.83**
*Syrphus ribesii*	93.10	0.07	4.33	0.04	4.00	0.00	118.80	0.09	27.86	0.03	124.05	**154.17**
*Lucilia caesar*	95.33	0.07	13.93	**0.22**	23.67	**0.15**	116.00	0.08	59.56	**0.10**	122.61	**187.50**
*Lucilia sericata*	95.11	0.06	16.00	0.03	0.00	0.00	53.00	0.04	24.50	0.03	109.03	**116.67**
*Eupeodes corollae*	97.75	0.05	2.00	0.03	66.00	0.08	56.60	0.04	13.00	0.01	101.79	**116.67**
*Eristalis tenax*	76.10	0.05	6.33	0.01	0.00	0.00	58.30	0.04	13.80	0.01	98.76	**116.67**
*Eristalis pertinax*	72.90	0.04	5.00	0.01	0.00	0.00	38.78	0.02	17.75	0.01	89.15	**104.17**
*Eristalis intricaria*	53.63	0.03	0.00	0.00	0.00	0.00	25.11	0.02	84.67	0.03	86.57	83.33
*Sphaerophoria scripta*	31.83	0.01	0.00	0.00	0.00	0.00	33.33	0.02	68.13	0.09	86.33	95.83
*Stomoxys calcitrans*	43.67	0.01	1.50	0.01	0.00	0.00	28.80	0.02	20.25	0.01	55.13	91.67
*Cerceris rybyensis*	36.80	0.01	0.00	0.00	0.00	0.00	9.00	0.00	1.00	0.00	45.11	33.33
*Cantharis pellucida*	39.33	0.01	0.00	0.00	0.00	0.00	11.22	0.01	40.00	0.01	37.00	54.17
*Bombus* sp.	24.20	0.02	2.25	0.01	8.50	0.03	28.20	0.02	6.56	0.01	33.83	**145.83**
*Bombus terrestris*	8.50	0.00	0.00	0.00	1.00	0.00	4.80	0.00	14.00	0.00	12.17	45.83
*Vespula vulgaris*	5.00	0.00	0.00	0.00	7.00	0.01	3.70	0.00	1.86	0.00	7.29	**112.50**
*Gasteruption assectator*	5.75	0.00	0.00	0.00	0.00	0.00	2.00	0.00	0.00	0.00	6.60	37.50
*Aglais io*	3.00	0.00	0.00	0.00	0.00	0.00	1.00	0.00	0.00	0.00	3.00	20.83
*Gaurotes virginea*	2.00	0.00	0.00	0.00	0.00	0.00	0.00	0.00	0.00	0.00	2.00	4.17
*Polistes dominula*	1.00	0.00	0.00	0.00	0.00	0.00	1.00	0.00	1.00	0.00	1.50	12.50
*Gasteruption* spp.	0.00	0.00	0.00	0.00	0.00	0.00	1.00	0.00	0.00	0.00	1.33	8.33
Community indices												
Site	1		2		3		4		5		Chi-Square	*p*
Total	1288.50	a	86.07	b	142.75	b	1362.10	a	639.22	Ab	33.02	0.0001
Species number	16.50	a **	5.80	b	6.00	b	18.40	a	12.67	Ab	37.88	0.0001
Berger-Parker dominance (D)	2.36	b	2.49	b	2.43	b	6.33	a	3.95	B	33.09	0.0001
Margalef (S)	1.50	ab	1.25	b	1.01	b	2.41	a	1.81	B	29.57	0.0001
$\mathrm{Shannon}-\mathrm{Weaver}\;(H^{\prime })$	1.23	b	1.40	b	1.41	b	2.43	a	1.97	Ab	33.63	0.0001
Pielou (J)	0.44	b	0.82	a	0.80	a	0.84	a	0.78	A	17.20	0.0018

The n-mean abundance of the species in a particular site; N—mean number of all taxa in a particular site; d-dominance index; Total—an abundance of the species in all stands; C-species stability index; Chi-square, *p*—results of Kruskal-Wallis test; * The bold values indicate the dominance D > 0.1 and the species stability C > 100; ** Different lowercase letters in rows indicate significant differences between treatments, Kruskal-Wallis test, *p* ≤ 0.05.

**Table 3:** The species similarity index between sites in 2019.

Site	1	2	3	4	5
1	x	0.52	0.50	0.92	0.86
2		x	0.62	0.52	0.88
3			x	0.41	0.50
4				x	0.92
5					x

**Table 4:** The species and community responses of pollinators observed on *Fallopia baldschuanica* flowers in 2020.

Site	1		2		3		4		5		Total	C
	N	d	n	d	n	d	n	d	n	d		
*Musca domestica*	170.10	**0.17**	233.40	**0.22**	212.58	**0.17**	119.33	0.07	115.09	**0.15**	171.65	150.72
*Episyrphus balteatus*	114.92	**0.17**	187.64	0.19	178.50	0.15	180.33	**0.16**	153.25	**0.23**	162.51	**159.46**
*Apis* spp.	94.75	**0.13**	179.18	**0.20**	193.42	**0.14**	140.75	**0.11**	35.58	0.04	127.88	**200.00**
*Sarcophaga* spp.	81.38	0.05	88.78	0.06	102.29	0.02	137.67	0.12	128.17	**0.20**	111.58	**135.21**
*Syrphus ribesii*	30.10	0.03	89.20	0.07	152.10	0.06	171.45	**0.11**	63.89	0.05	103.50	**126.58**
*Lucilia caesar*	62.08	0.08	137.56	**0.11**	100.55	0.07	98.73	0.09	58.80	0.06	89.87	158.21
*Sphaerophoria scripta*	5.20	0.00	8.67	0.00	122.88	0.04	167.33	0.08	62.09	0.06	83.33	**100.00**
*Lucilia sericata*	54.33	0.09	84.90	0.08	69.10	0.04	126.60	0.07	52.57	0.03	78.08	**144.12**
*Eupeodes corollae*	40.83	0.02	3.88	0.00	150.75	0.06	5.00	0.00	44.40	0.02	61.04	76.71
*Chrysotoxum bicinctum*	95.70	**0.11**	4.60	0.00	58.42	0.07	70.00	0.07	8.67	0.00	60.64	**116.67**
*Eristalis tenax*	32.50	0.03	8.90	0.01	82.27	0.04	72.71	0.03	55.92	0.06	49.31	**136.84**
*Eristalis pertinax*	13.63	0.01	2.83	0.00	90.00	0.04	38.86	0.01	48.78	0.03	43.43	**103.90**
*Eristalis intricaria*	29.33	0.02	19.71	0.02	63.64	0.03	50.00	0.01	19.11	0.02	36.51	**104.00**
*Bombus* sp.	18.64	0.02	11.09	0.01	50.67	0.03	60.25	0.05	20.42	0.02	32.81	**193.33**
*Stomoxys calcitrans*	47.25	0.02	4.00	0.00	25.88	0.01	76.00	0.00	54.33	0.01	31.56	77.14
*Polistes dominula*	5.43	0.00	8.86	0.01	23.56	0.01	4.00	0.00	12.50	0.00	13.07	84.85
*Vespula vulgaris*	7.17	0.01	10.27	0.01	9.80	0.00	4.55	0.00	4.40	0.00	7.24	**166.15**
*Bombus terrestris*	5.00	0.00	4.20	0.00	8.33	0.00	11.13	0.00	6.00	0.00	6.88	**108.20**
*Cantharis pellucida*	4.00	0.00	2.40	0.00	12.86	0.00	0.00	0.00	5.00	0.00	6.50	55.00
*Cerceris rybyensis*	4.50	0.00	7.38	0.01	5.00	0.00	4.00	0.00	2.00	0.00	5.46	77.42
*Gaurotes virginea*	3.00	0.00	1.25	0.00	5.17	0.00	14.00	0.00	0.00	0.00	3.89	46.91
*Gasteruption assectator*	2.00	0.00	1.33	0.00	0.00	0.00	2.00	0.00	1.00	0.00	1.57	22.22
*Aglais io*	1.50	0.00	1.50	0.00	1.00	0.00	2.00	0.00	0.00	0.00	1.40	24.39
*Gasteruption* spp.	1.25	0.00	1.25	0.00	1.00	0.00	0.00	0.00	0.00	0.00	1.18	34.38
Community indices												
Site	1		2		3		4		5		Chi-Square	*p*
Total	784.58	b	995.64	b	1481.25	a	1255.25	a	777.50	B	33.47	0.0001
Species number	17.25	a	16.73	a	17.00	a	12.75	b	12.92	B	37.88	0.0001
Berger-Parker dominance (d)	2.69	b	3.88	ab	5.26	a	5.58	a	4.42	ab	33.09	0.0001
Margalef (S)	1.66	b	2.28	a	2.26	a	1.70	b	1.83	B	29.57	0.0001
$\mathrm{Shannon}-\mathrm{Weaver}\;\left(H^{\prime }\right)$	1.37	b	1.90	ab	2.33	a	2.21	a	2.03	ab	33.63	0.0001
Pielou (J)	1.37	b	1.90	ab	2.33	a	2.21	a	2.03	ab	33.63	0.0001
Total	0.48	b	0.69	ab	0.83	a	0.87	a	0.80	ab	17.20	0.0017

The n-mean abundance of the species in a particular site; N—mean number of all taxa in a particular site; d-dominance index; Total—an abundance of the species in all stands; C-species stability index; Chi-square, *p*—results of Kruskal-Wallis test; The bold values indicate the dominance D > 0.1 and the species stability C > 100; Different lowercase letters in rows indicate significant differences between treatments, Kruskal-Wallis test, *p* ≤ 0.05.

**Table 5:** The species similarity index between sites in 2020.

	1	2	3	4	5
1	x	1	0.96	0.92	0.86
2		x	0.96	0.92	0.92
3			x	0.96	0.83
4				x	0.87
5					x

## Data Availability

The data sets generated and analyzed in the present study may be available from the corresponding author upon request.

## Supplementary Materials

The following supporting information can be downloaded at: https://www.mdpi.com/article/10.3390/insects13100904/s1, Supplementary Materials S1: Comparison of the identified organic compounds (VOCs) with the mass spectral library. Supplementary Materials S2: The description of ecological indices used for data analysis. Table S1: List of floral visitors and true pollinators of *Fallopia baldschuanica* in Wrocław, SW Poland. Table S2: The statistics of the Canonical Correspondence Analysis (CCA) for 2019. Table S3: The statistics of the Canonical Correspondence Analysis (CCA) for 2020. Figure S1: The CCA biplot shows the taxa abundance in relation to the date and time of observations in 5 sites in 2019. Figure S2: The CCA biplot shows the taxa abundance in relation to the date and time of observations in 5 sites in 2020.
