# Supplementary material for: Floral Volatile Organic Compounds and a List of Pollinators of Fallopia baldschuanica (Polygonaceae)
Source: Insects. 2022 Oct 5;13(10):904. doi: 10.3390/insects13100904 (PMC9604458; doi:10.3390/insects13100904)
Supplement: Supplementary file 1 [file insects-13-00904-s001.zip › Supplementary Figures and Tables.pdf]

Supplementary Figure S1. The CCA biplot shows the taxa abundance in relation to the date and time of observations in 5 sites in 2019.

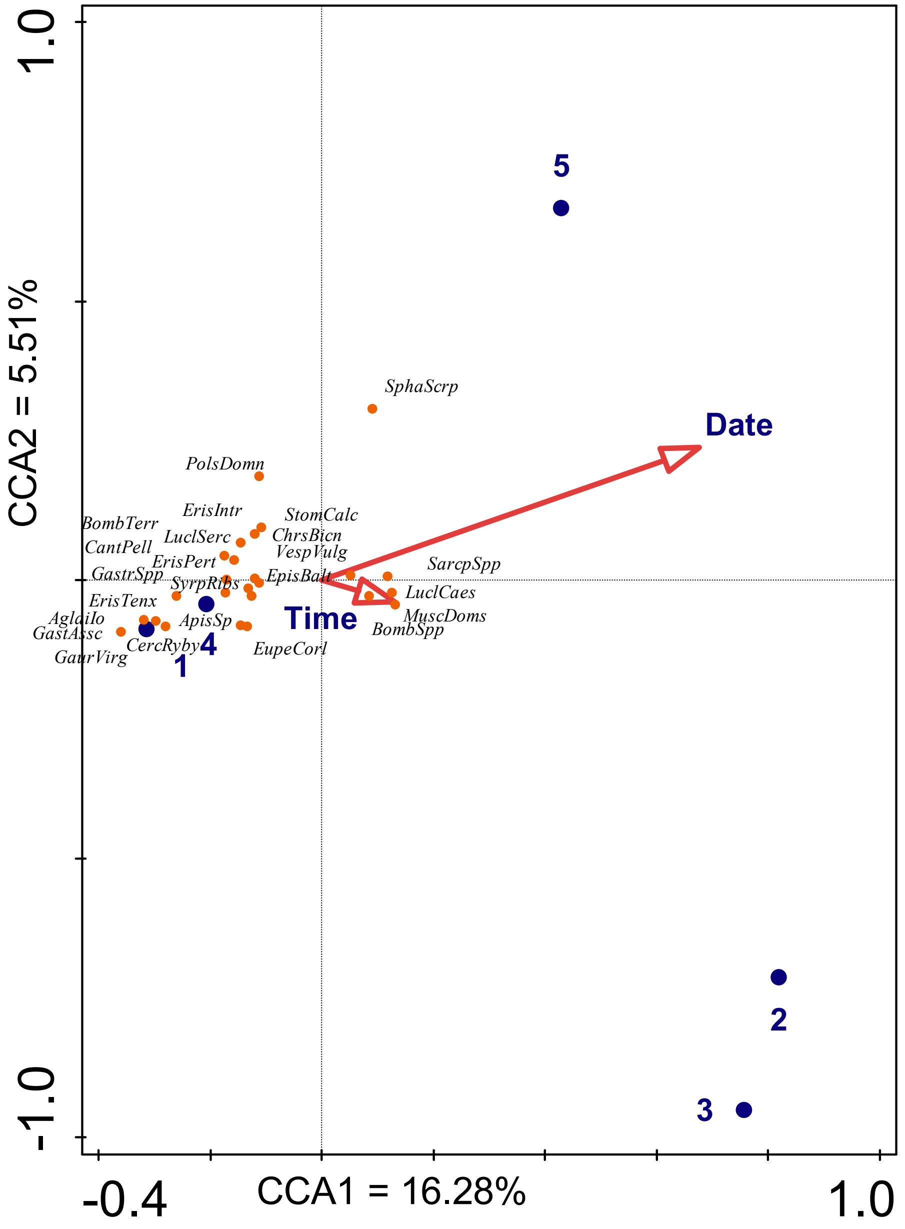

The full species name is given in Table S1;

Date – study period from 5.08.2019-24.10.2020;

Time – time of the day: 9:00 a.m. - 6:00 p.m.

Supplementary Figure S2. The CCA biplot shows the taxa abundance in relation to the date and time of observations in 5 sites in 2020.

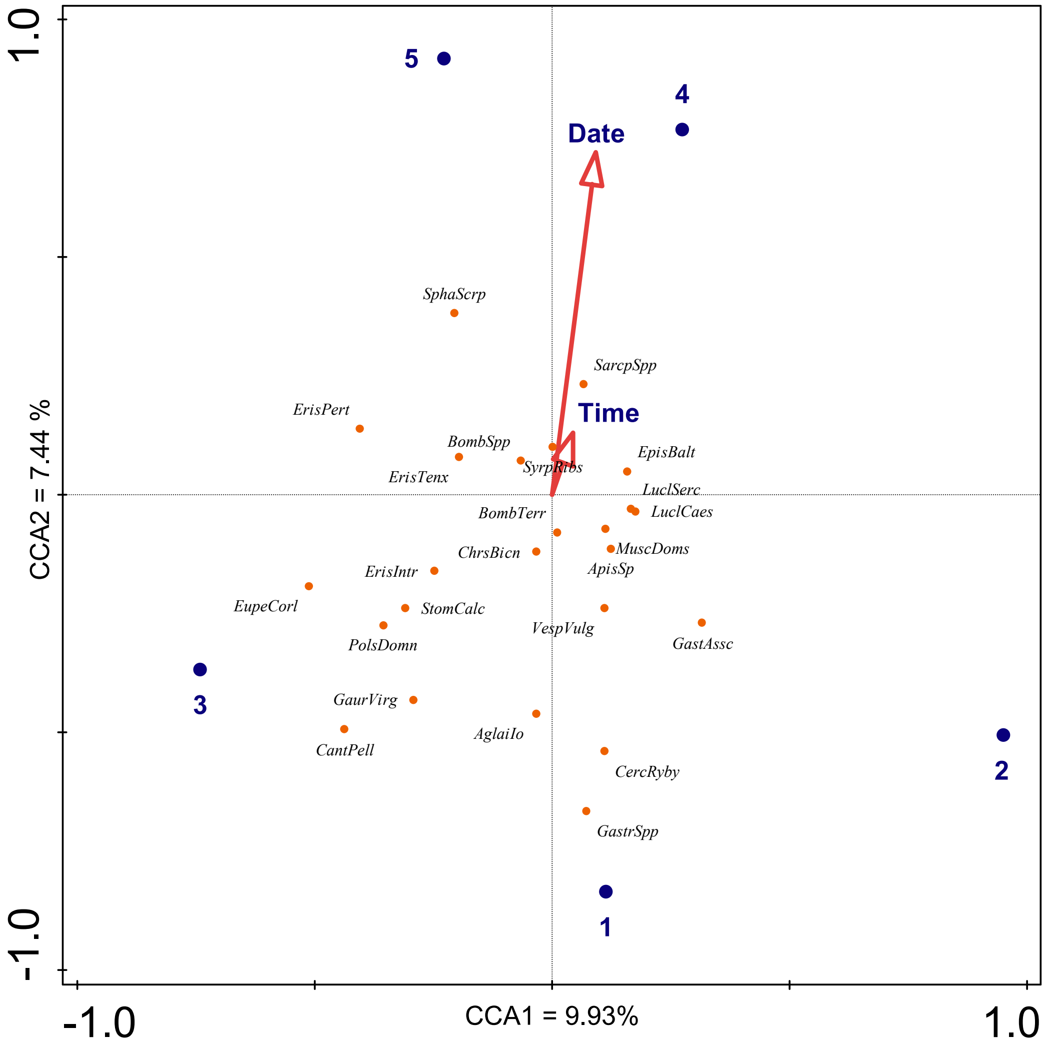

The full species name is given in Table S1;

Date – study period from 5.08.2019-24.10.2020;

Time – time of the day: 9:00 a.m. - 6:00 p.m.

Supplementary Table S1. List of floral visitors and true pollinators of *Fallopia baldschuanica* in Wrocław, SW Poland.

**Insect order**	**Family**	**Species**	**Abbreviation**
Hymenoptera	Apidae	*Apis sp.**	*ApisSp*
		Bombus sp.*	*BombSpp*
		*Bombus terrestris**	*BombTerr*
	Crabronidae	*Cerceris rybyensis**	*CercRyby*
	Gasteruptiidae	*Gasteruption assectator**	*GastAssc*
		Gasteruption spp.*	*GastrSpp*
	Vespidae	*Vespula vulgaris**	*VespVulg*
		*Polistes dominula**	*PolsDomn*
Diptera	Calliphoridae	*Lucilia caesar**	*LuclCaes*
		*Lucilia sericata**	*LuclSerc*
	Muscidae	*Musca domestica**	*MuscDoms*
		*Stomoxys calcitrans**	*StomCalc*
	Sarcophagidae	*Sarcophaga* spp.*	*SarcpSpp*
	Syrphidae	*Chrysotoxum bicinctum**	*ChrsBicn*
		*Eupeodes corollae**	*EupeCorl*
		*Episyrphus balteatus**	*EpiBalt*
		*Eristalis intricaria**	*ErisIntr*
		*Eristalis tenax**	*ErisTenx*
		*Eristalis pertinax**	*ErisPert*
		*Sphaerophoria scripta**	*SphaScrp*
		*Syrphus ribesii**	*SyprRibs*
Coleoptera	Cantharidae	*Cantharis pellucida*	*CantPell*
	Cerambycidae	*Gaurotes virginea*	*GaurVirg*
Lepidoptera	Nymphalidae	*Aglais io*	*AglaiIo*

Abbreviation: * true pollinators

Supplementary Table S2. The statistics of the Canonical Correspondence Analysis (CCA) for 2019.

	**CCA1**	**CCA2**	**CCA3**	**CCA4**
Eigenvalues	0.094	0.031	0.0092	0.0067
Explained variation (cumulative)	16.28	21.59	23.18	24.34
Pseudo-canonical correlation	0.85	0.79	0.54	0.50
Total variation	0.58			
Explanatory variables variance	25.24%			
Significance of the axes	F=19.4, P=0.002			

Supplementary Table S3. The statistics of the Canonical Correspondence Analysis (CCA) for 2020.

	**CCA1**	**CCA2**	**CCA3**	**CCA4**
Eigenvalues	0.074	0.055	0.034	0.019
Explained variation (cumulative)	9.93	17.37	21.91	24.45
Pseudo-canonical correlation	0.82	0.83	0.77	0.63
Total variation	0.74			
Explanatory variables variance	26.18%			
Significance of the axes	F=27.3, P=0.002			
