# Supplementary material for: Floral Volatile Organic Compounds and a List of Pollinators of Fallopia baldschuanica (Polygonaceae)
Source: Insects. 2022 Oct 5;13(10):904. doi: 10.3390/insects13100904 (PMC9604458; doi:10.3390/insects13100904)
Supplement: Supplementary file 1 [file insects-13-00904-s001.zip › Supplementary Materials S1 and S2.pdf]

Supplementary Material S1. Comparison of the identified organic compounds (VOCs) with the mass spectral library.

Numbers 1-18 correspond with Table 1.

1.

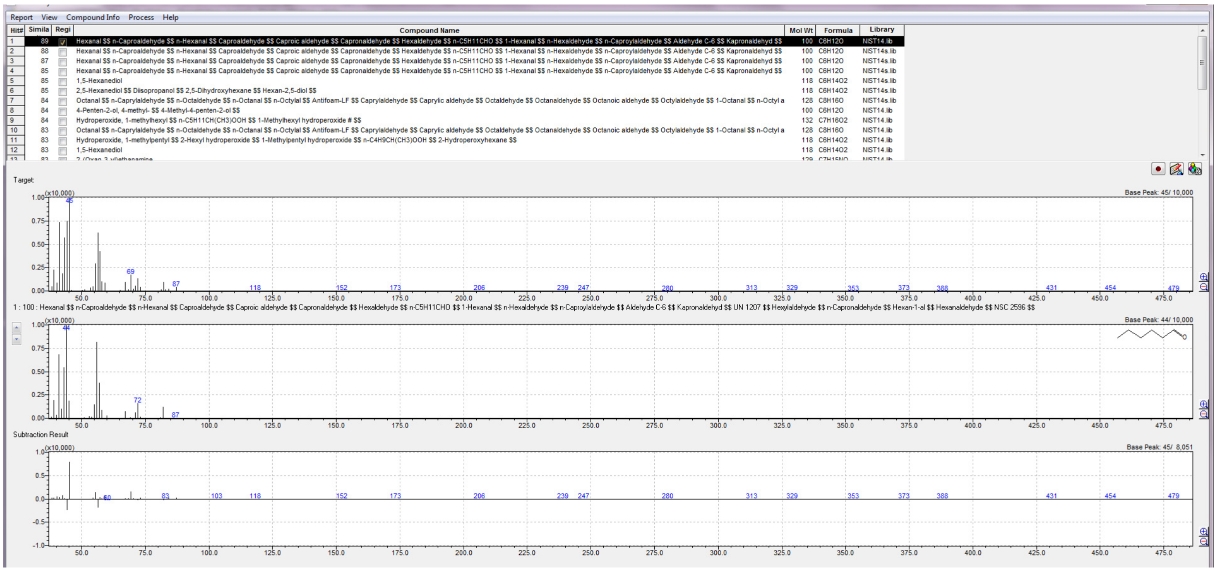

2.

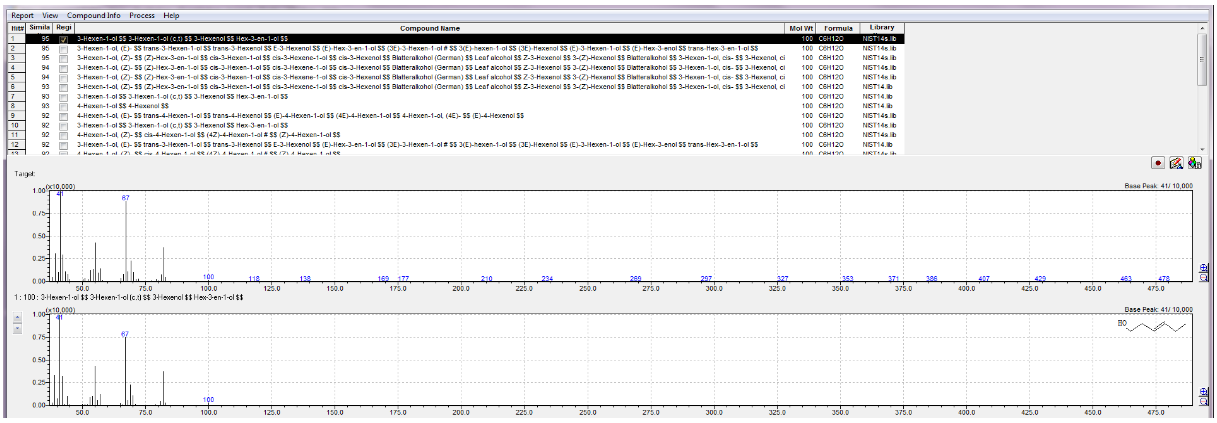

3.

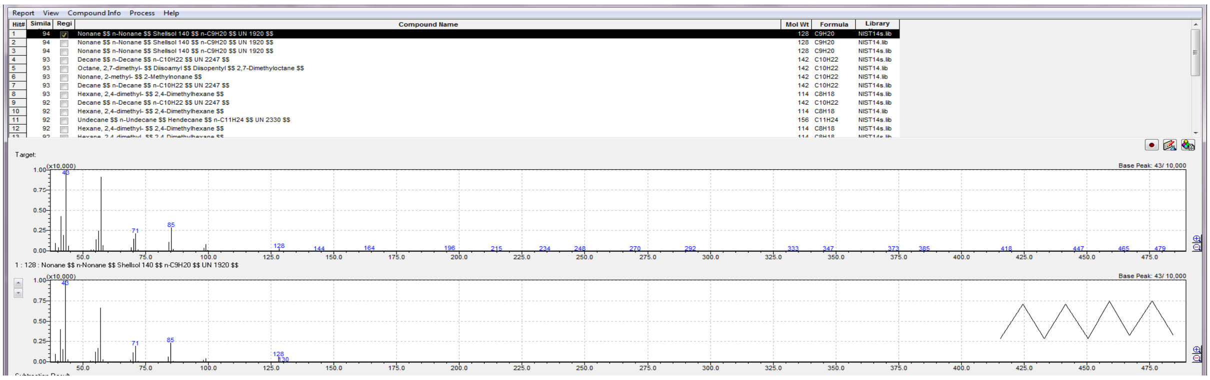

4.

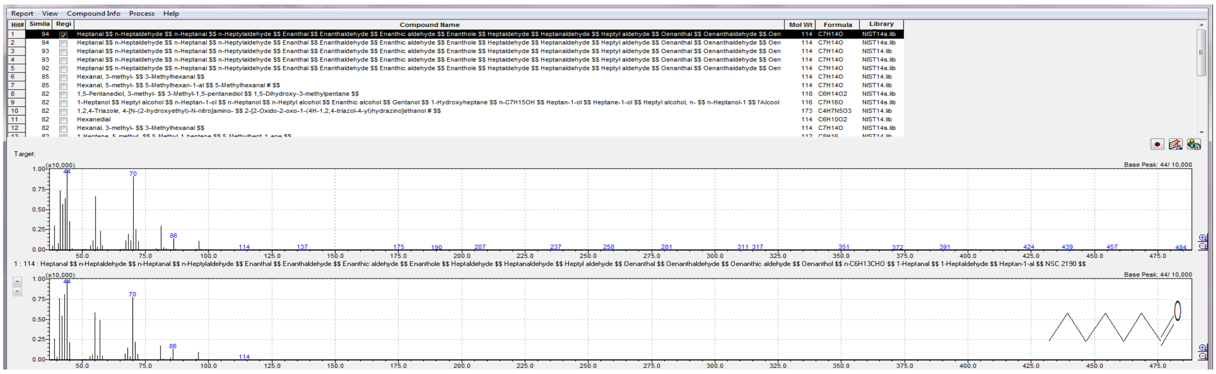

5.

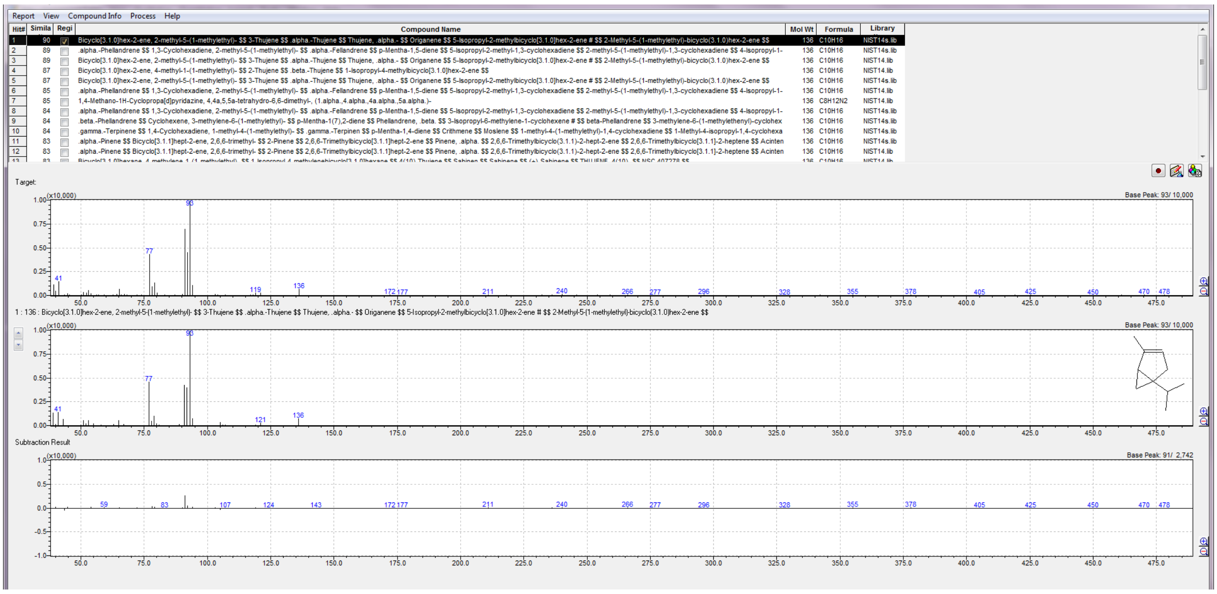

6.

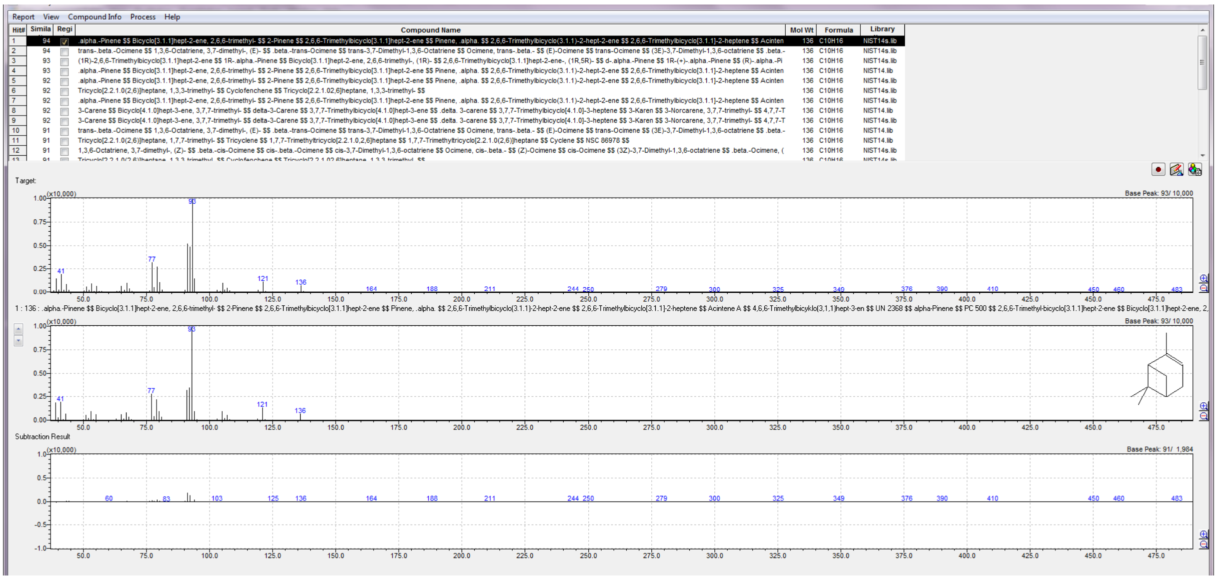

7.

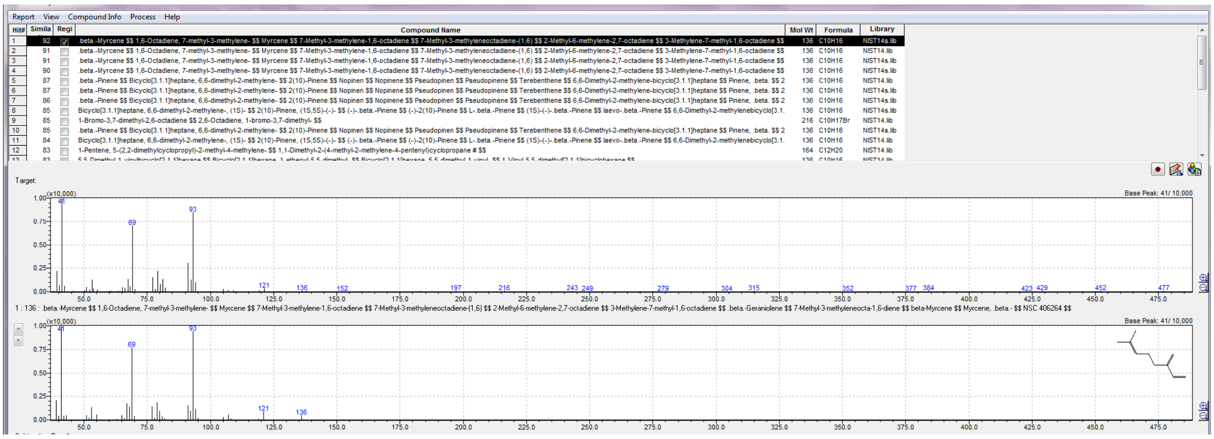
Report View Compound Info Process Help
Hit# Simila Regi Compound Name Mol Wt Formula Library
Target:
Base Peak: 41/ 10,000

8.

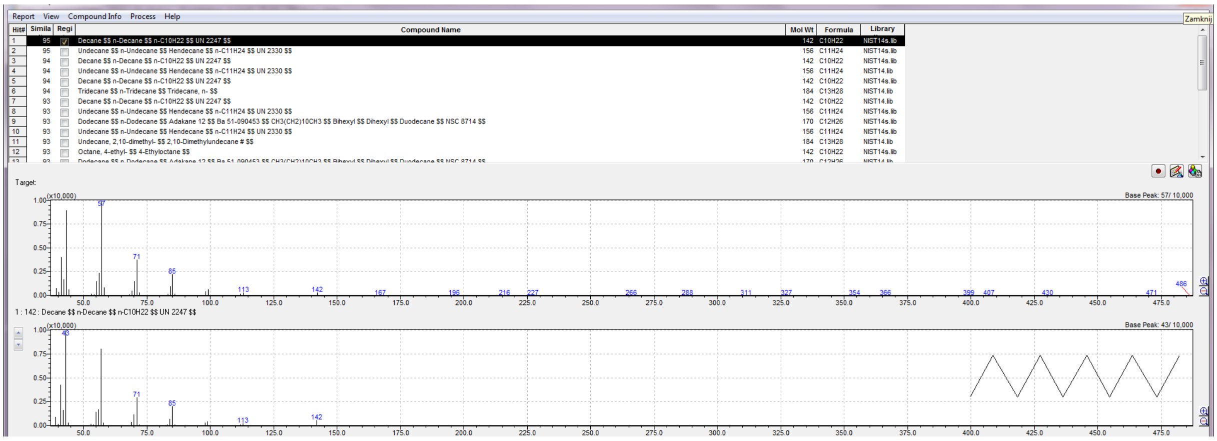
Report View Compound Info Process Help
Hit# Simila Regi Compound Name Mol Wt Formula Library
Decane $$ n-Decane $$ n-C10H22 $$ UN 2247 $$
Target:
Base Peak: 57/ 10,000
1 : 142 : Decane $$ n-Decane $$ n-C10H22 $$ UN 2247 $$
Base Peak: 43/ 10,000

9.

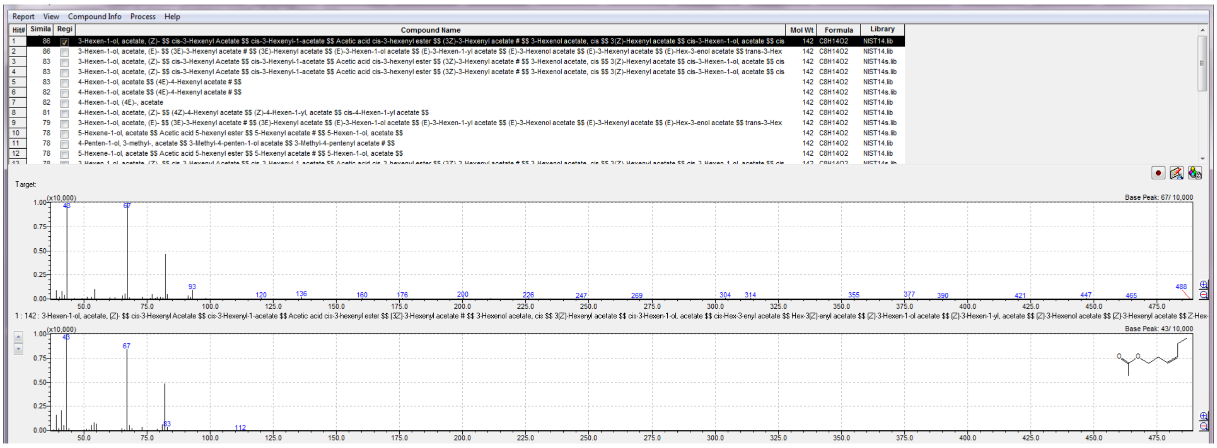
Report View Compound Info Process Help
Hit# Simila Regi Compound Name Mol Wt Formula Library
Target:
Base Peak: 67/ 10,000
Base Peak: 43/ 10,000

10.

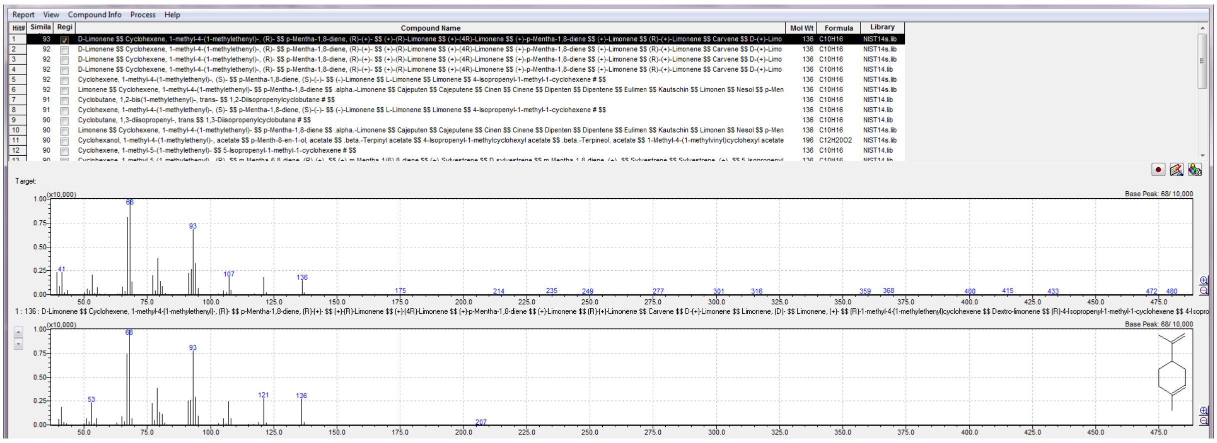

11.

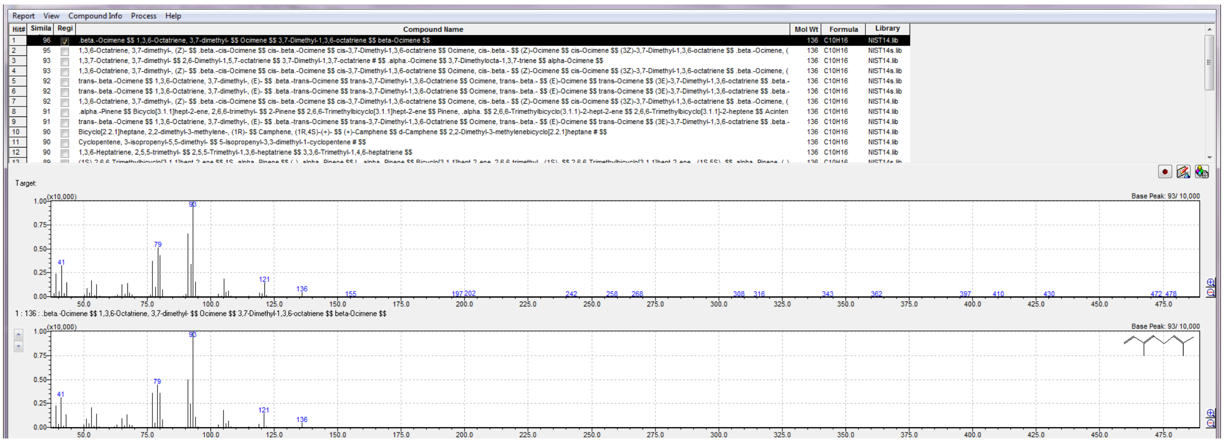

12.

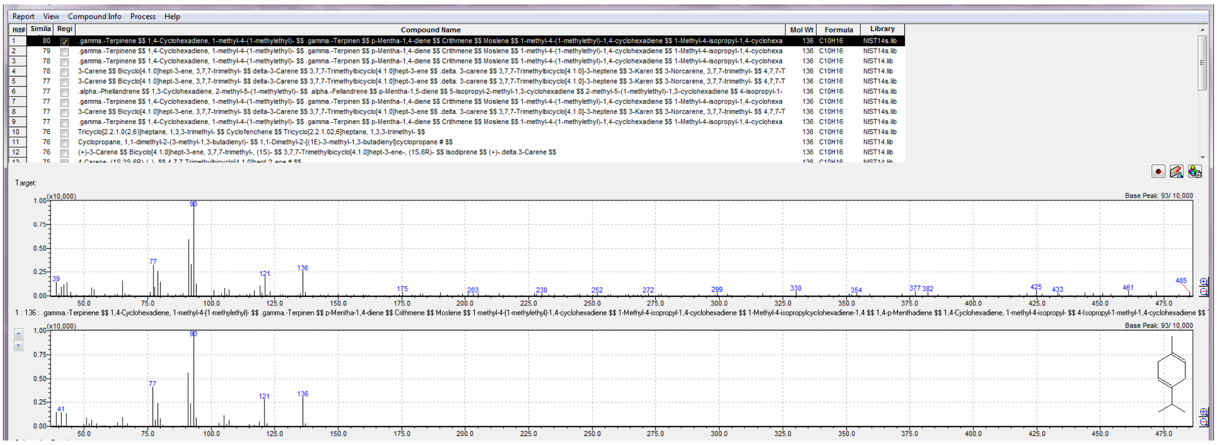

13.

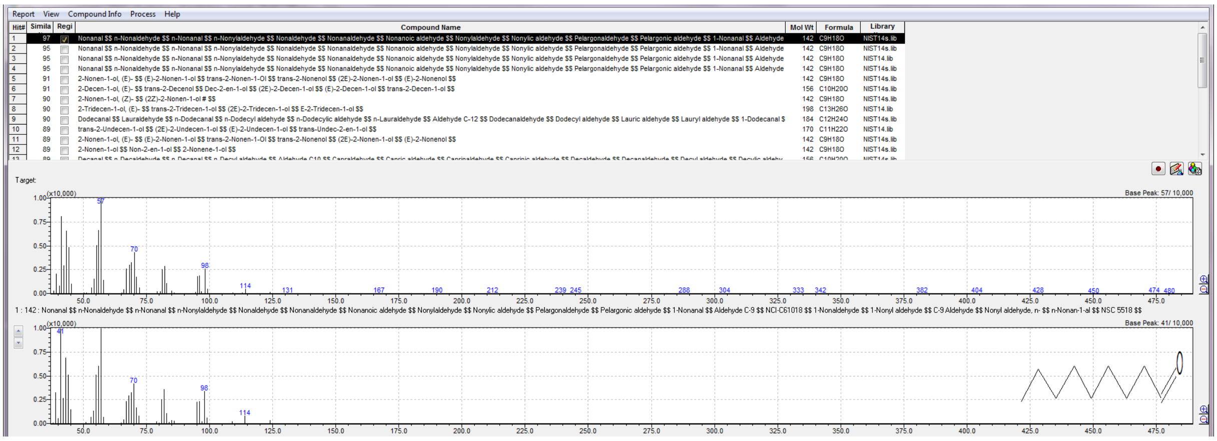
1 : 142 : Nonanal $$ n-Nonaldehyde $$ n-Nonanal $$ n-Nonylaldehyde $$ Nonaldehyde $$ Nonanaldehyde $$ Nonanoic aldehyde $$ Nonylaldehyde $$ Nonylic aldehyde $$ Pelargonaldehyde $$ Pelargonic aldehyde $$ 1-Nonanal $$ Aldehyde C-9 $$ NCI-C61018 $$ 1-Nonaldehyde $$ 1-Nonyl aldehyde $$ C-9 Aldehyde $$ Nonyl aldehyde, n- $$ n-Nonan-1-al $$ NSC 5518 $$

14.

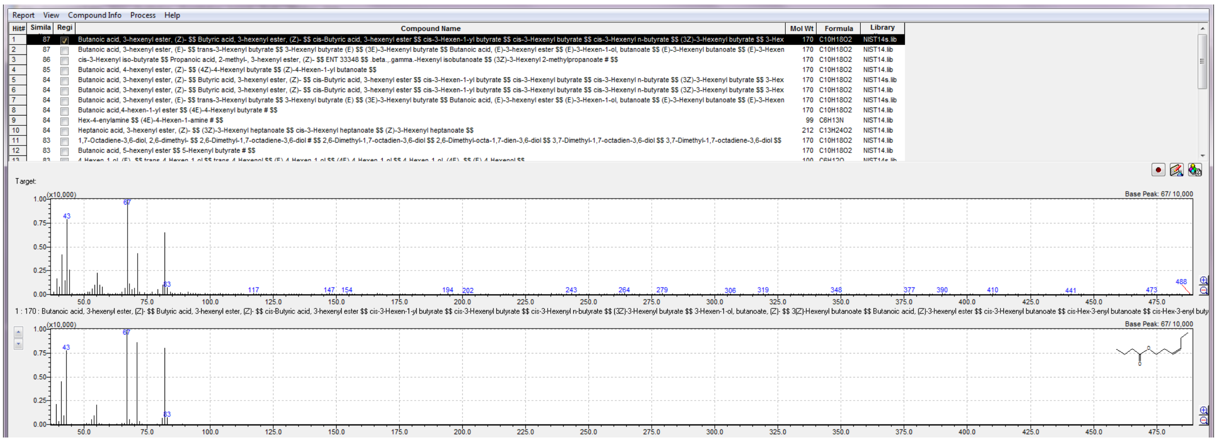
1 : 170 : Butanoic acid, 3-hexenyl ester, (Z)- $$ Butyric acid, 3-hexenyl ester, (Z)- $$ cis-Butyric acid, 3-hexenyl ester $$ cis-3-Hexen-1-yl butyrate $$ cis-3-Hexenyl butyrate $$ cis-3-Hexenyl n-butyrate $$ (3Z)-3-Hexenyl butyrate $$ 3-Hexen-1-ol, butanoate, (Z)- $$ 3(Z)-Hexenyl butanoate $$ Butanoic acid, (Z)-3-hexenyl ester $$ cis-3-Hexenyl butanoate $$ cis-Hex-3-enyl butanoate $$ cis-Hex-3-enyl buty

15.

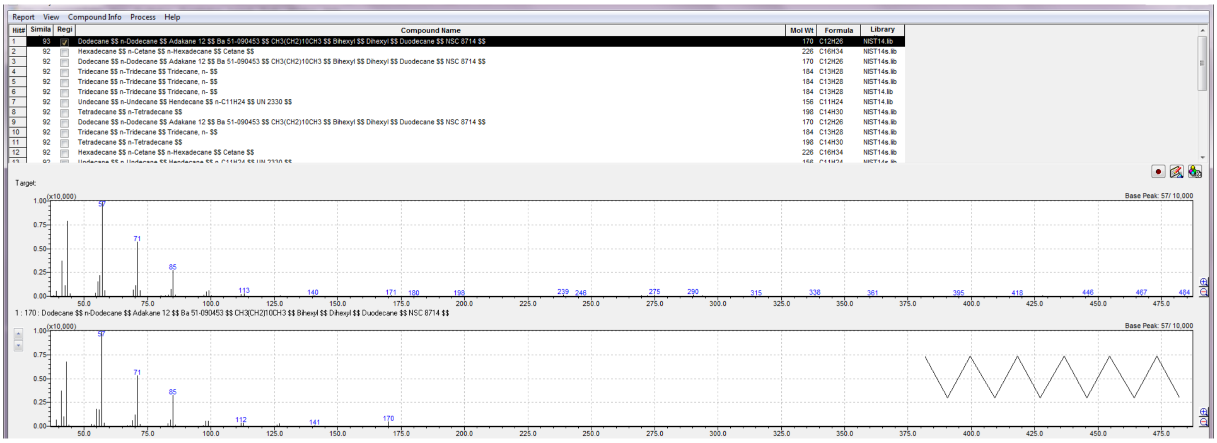
1 : 170 : Dodecane $$ n-Dodecane $$ Adakane 12 $$ Ba 51-090453 $$ CH3(CH2)10CH3 $$ Bihexyl $$ Dihexyl $$ Duodecane $$ NSC 8714 $$

16.

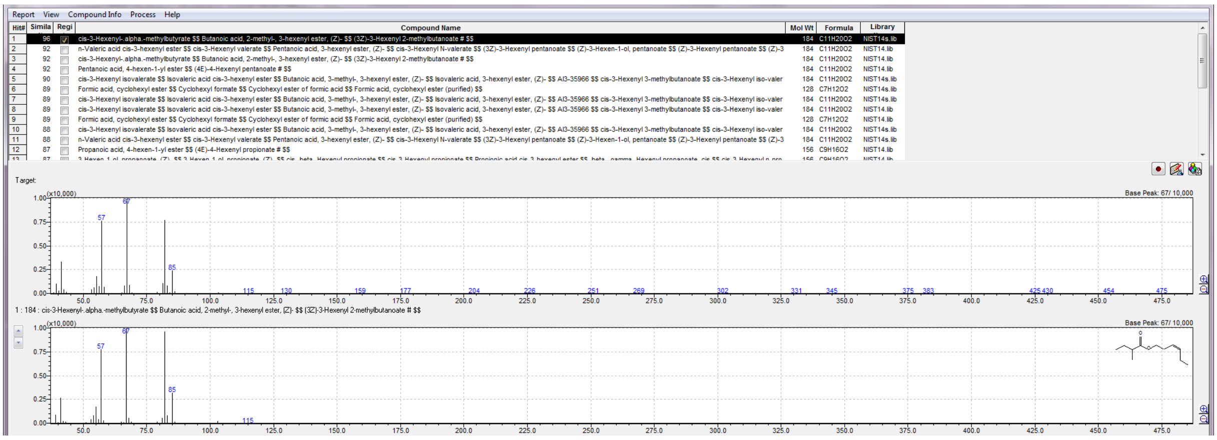

Report View Compound Info Process Help
Target:
Base Peak: 67/ 10,000
1 : 184 : cis-3-Hexenyl-.alpha.-methylbutyrate $$ Butanoic acid, 2-methyl-, 3-hexenyl ester, (Z)- $$ (3Z)-3-Hexenyl 2-methylbutanoate # $$

17.

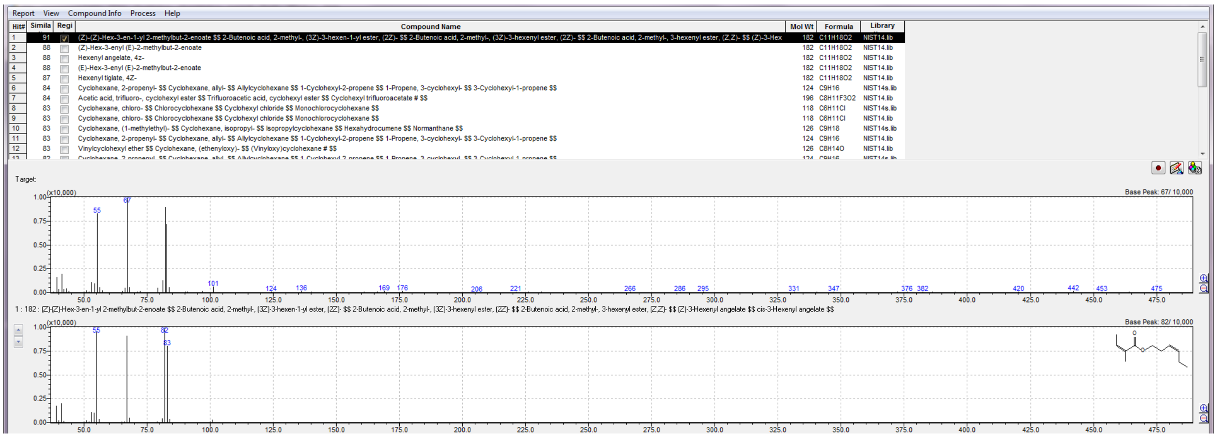

Report View Compound Info Process Help
Target:
Base Peak: 67/ 10,000
Base Peak: 82/ 10,000

18.

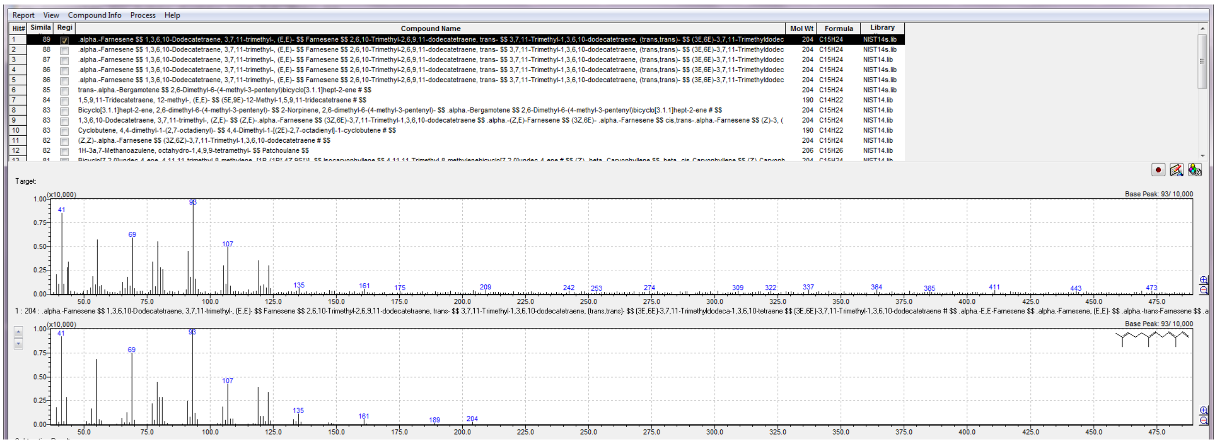

Report View Compound Info Process Help
Target:
Base Peak: 93/ 10,000

Supplementary material S2. The description of ecological indices used for data analysis.

The **dominance index (*d*)** of each species was calculated according to the formula:

$$d = \frac{\mathrm{n}_i}{\mathrm{N}}$$

where n_i is the abundance of individuals belonging to the ith taxa and N – the total abundance of all taxa.

The **Berger-Parker dominance index (*D*)** was calculated according to the formula:

$$D = \frac{\mathrm{n}_{max}}{\mathrm{N}}$$

where n_{max} is the abundance of the most abundant species taxa and N – is the total abundance of all taxa.

The **Shannon-Weaver (*H'*)** index was calculated according to the following formula:

$$H' = -\sum_{i=1}^{R} \mathrm{D}\ \mathrm{ln}d$$

where p_i is the proportion of individuals belonging to the ith taxa.

The **Pielou (*J*)** index was calculated according to the formula:

$$J = \frac{\mathrm{H'}}{\mathrm{ln}(S)}$$

where H' is the Shannon-Weaver index and *S* is the total number of species in a sample.

The **Margalef's species richness (*S*)** index was calculated according to the following formula:

$$S = \frac{s - 1}{\mathrm{ln}(N)}$$

where *s* is the total number of species in a sample and *N* the total number of individuals in the sample.

The **species stability (*C*)** was calculated according to the following formula:

$$C = 200\frac{n_a}{\mathrm{N}}$$

where n_a is the total number of samples with species and N is the total number of samples.

The **Jaccard similarity index (*SJ*)** was calculated according to the formula:

$$SJ = \frac{C}{\mathrm{a} + \mathrm{b} + \mathrm{c}}$$

where c is the number of shared species between the two sites and a and b are the number of species unique to each site.

References

Berger, W.H., Parker F.L. Diversity of Planktonic Foraminifera in Deep Sea Sediments. *Science* **1970**, 168, 1345. https://doi.org/10.1126/science.168.3937.1345.

Shannon, C.E. A Mathematical Theory of Communication. *Bell Syst. Tech. J.* **1948**, 27, 379-423. https://doi.org/10.1002/j.1538-7305.1948.tb01338.x

Pielou, E.C. The measurement of diversity in different types of biological collections. *J. Theor. Biol.* **1966**, 13, 131-144. https://doi.org/10.1016/0022-5193(66)90013-0

Margalef, R., Information Theory in Ecology. *General Systems* **1958**, 3, 36-71.

Trojan, P. *General Ecology (Ekologia ogólna)*; PWN Publishing: Warsaw, Poland, 1980.

Jaccard, P. The Distribution of the Flora of the Alpine Zone. *New Phytologist* **1912**, 11, 37-50. http://dx.doi.org/10.1111/j.1469-8137.1912.tb05611.x
